# Decreased Mitochondrial DNA Mutagenesis in Human Colorectal Cancer

**DOI:** 10.1371/journal.pgen.1002689

**Published:** 2012-06-07

**Authors:** Nolan G. Ericson, Mariola Kulawiec, Marc Vermulst, Kieran Sheahan, Jacintha O'Sullivan, Jesse J. Salk, Jason H. Bielas

**Affiliations:** 1 Molecular Diagnostics Program, Division of Public Health Sciences, Fred Hutchinson Cancer Research Center, Seattle, Washington, United States of America; 2 Department of Chemistry, University of North Carolina, Chapel Hill, North Carolina, United States of America; 3 St. Vincent's University Hospital, Dublin, Ireland; 4 Department of Pathology, University of Washington, Seattle, Washington, United States of America; Baylor College of Medicine, United States of America

## Abstract

Genome instability is regarded as a hallmark of cancer. Human tumors frequently carry clonally expanded mutations in their mitochondrial DNA (mtDNA), some of which may drive cancer progression and metastasis. The high prevalence of clonal mutations in tumor mtDNA has commonly led to the assumption that the mitochondrial genome in cancer is genetically unstable, yet this hypothesis has not been experimentally tested. In this study, we directly measured the frequency of non-clonal (random) de novo single base substitutions in the mtDNA of human colorectal cancers. Remarkably, tumor tissue exhibited a decreased prevalence of these mutations relative to adjacent non-tumor tissue. The difference in mutation burden was attributable to a reduction in C∶G to T∶A transitions, which are associated with oxidative damage. We demonstrate that the lower random mutation frequency in tumor tissue was also coupled with a shift in glucose metabolism from oxidative phosphorylation to anaerobic glycolysis, as compared to non-neoplastic colon. Together these findings raise the intriguing possibility that fidelity of mitochondrial genome is, in fact, increased in cancer as a result of a decrease in reactive oxygen species-mediated mtDNA damage.

## Author Summary

Mitochondria are the intracellular organelles responsible for energy production in eukaryotic cells. They are unique in that they contain their own DNA (mtDNA), which encodes genes important for mitochondrial function and is the cell's only genetic material stored outside the nucleus. Mutations in both nuclear and mtDNA are believed to have a role in tumor growth and metastasis. While it is well established that nuclear DNA has an increased overall burden of mutations in human cancers, this has not been investigated in mtDNA. Here we use a highly sensitive assay to determine mutational load in mtDNA isolated from patient-matched normal and cancerous colonic tissues. Surprisingly, we discovered that the frequency of mutations in mtDNA is actually decreased in tumors relative to healthy tissues, suggesting that, unlike in nuclear DNA, accelerated mutagenesis in mtDNA does not facilitate a cancer's development and may even hinder it. These findings raise the possibility that mitochondrial-targeted cancer therapeutics focused on directly increasing mtDNA damage and mutation might effectively suppress malignant growth.

## Introduction

Genetic heterogeneity is an important feature of human cancers. The ongoing introduction of rare somatic mutations into the genome of each cell within a developing tumor provides the necessary genetic diversity to fuel the adaptive evolution that drives disease progression [1]. Among the many random mutations that arise in an evolving cancer, only a small fraction will confer their host cell with a neoplastic advantage. Those that do, however, may undergo positive selection and clonally proliferate until they, and their resulting phenotype, drive continued tumor progression.

A preponderance of evidence points to the importance of acquired genetic instability in the nuclear genome as a key facilitator of tumorigenesis [2]. Far less attention, however, has been paid to alterations in replication fidelity of the mitochondrial genome. Mitochondria are semi-autonomous entities with a unique biology whose genomic replication is independent of the cell cycle and accomplished with a distinct complement of enzymatic machinery [3]. Over the last decade, multiple sequencing efforts have revealed that the mitochondrial genomes of human tumors frequently carry clonally expanded mtDNA mutations [4], [5]. Mounting evidence indicates that a subset of these mutations directly contributes to cancer progression by accelerating primary tumor growth [6] and conferring metastatic potential [7] to tumor cells.

An open question remains as to whether the nuclear point mutation instability of human cancers [8] is recapitulated in the mitochondrial genome. Understanding mitochondrial mutagenesis in normal and tumor cells will further delineate a fundamental process in cancer progression and potentially identify novel mitochondrial targets for cancer prevention, treatment and early diagnosis. In this study, we address this question by using the high-sensitivity of the Random Mutation Capture (RMC) assay [9], [10] to directly measure the frequency of non-clonal (random) mtDNA mutations in normal and colorectal cancer tissues.

## Results

Clonal mutations represent identical mutant mtDNA molecules that are present in the majority of genomes within a cell population. Such mutations occur via propagation of the genotype of a single mutant genome in a single founder cell to all cellular descendants during clonal proliferation. In a tumor, clonal mutations reflect the genotype of the founding cell of the terminal clonal outgrowth. Random mutations, in contrast, are mutations that arise in cell divisions after the founding of a clonal population and are present in only a subset of cells. The frequency of random mutations in a population is proportional to the rate of mutation and dependent on the number of cell divisions having led to the generation of the sampled population.

First, to stratify our colorectal tissue samples with respect to the abundance of clonally expanded mutations, we sequenced the entire mitochondrial genome of each of our samples. We found that 55% (11 of 20) of the carcinomas carried at least one clonally expanded mutation in their mtDNA (Table 1). Furthermore, when located inside a protein-coding gene, the mutations identified in our tumors uniformly resulted in frameshift mutations (2/13) or non-synonymous changes (11/13). Although the observed frequency of non-synonymous point mutations (11/11) exceeded that expected by chance, the difference did not reach significance in our sample set (P = 0.06, 2-tailed chi-square test, n = 11). To assess whether the expansion of mtDNA mutations is an early or a late event during carcinogenesis, we also sequenced the entire mitochondrial genome of 19 patient-matched adenomas. We found that 32% (6 of 19) of the adenomas carried clonally expanded mutations (Table 1), revealing that clonal expansion of mtDNA mutations can occur prior to overt malignancy.

**Table 1:** Clonal Mitochondrial DNA Mutations Identified in Colorectal Tissue.

Patient ID	Tissue	Gene	DNA	Protein
2	Carcinoma	COXII	7859G>A	D92N
		COXIII	9451G>A	G82E
4	Carcinoma	non-coding	5894insC	
		Cyt b	15375G>A	G210E
5	Carcinoma	D-loop	508A>G	
6	Carcinoma	COXIII	9645G>A	A147T
7	Carcinoma	D-loop	523delAC	
		12S rRNA	1598G>A	
		ND2	4707C>T	L80F
10	Carcinoma	ND4L	10675G>A	C69Y
		ND5	13239C>A	I301M
		D-loop	16465C>T	
11	Carcinoma	D-loop	72T>C	
		D-loop	309insC	
		COXI	6121T>C	I73T
		COXIII	9439G>A	G78D
17	Carcinoma	ND5	12426insA	frameshift
		D-loop	16298C>T	
		D-loop	16390G>A	
18	Carcinoma	ND1	3745G>A	A147T
		ND5	13133delT	frameshift
19	Carcinoma	ND5	13970G>A	S545N
20	Carcinoma	D-loop	524insAC	
		16S rRNA	2957G>A	
18	Adenoma	ND5	13193T>C	L286P
19	Adenoma	D-loop	66delG	
		ND1	4142G>A	R279Q
		COXII	8251G>A	
		ND4	11711G>A	A318T
		D-loop	16183insC	
20	Adenoma	D-loop	524insAC	
		16S rRNA	2957G>A	
69	Adenoma	ND1	3571insC	frameshift
		ND2	5450C>T	
70	Adenoma	tRNA M	4465T>C	
		Cyt b	15448C>T	
74	Adenoma	12S rRNA	1045G>A	
		COXII	8141G>A	A186T
		ND4	10952insC	frameshift
		D-loop	16166A>G	
		D-loop	16309A>G	

**Figure 1:**
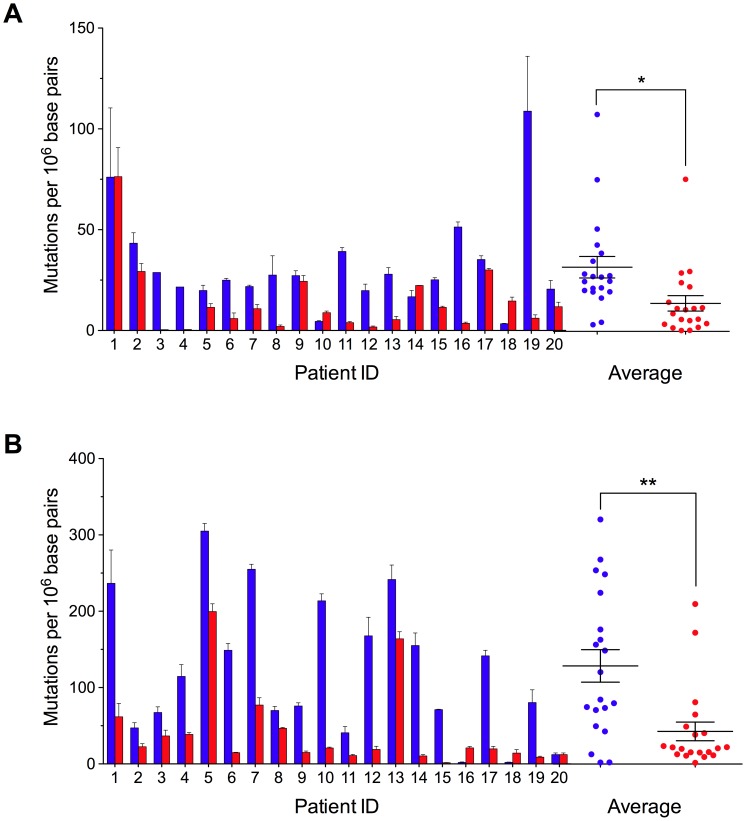
Decreased Random Mitochondrial DNA Mutations in Colorectal Cancer. (A) Mutation frequency (± s.e.m.) was determined at *Taq*I restriction sites 1215–1218 within the 12S rRNA gene and (B) 7335–7338 within the COXI gene in mitochondrial DNA isolated from patient-matched normal (blue) and carcinoma (red) colorectal tissues. The mean (n = 20) mutation burden (± s.e.m.) of mtDNA isolated from carcinoma versus normal tissue is reduced ∼3-fold at both mutational target sites. * *P*<0.01; ** *P*<0.001; two-tailed paired t-test.

**Figure 2:**
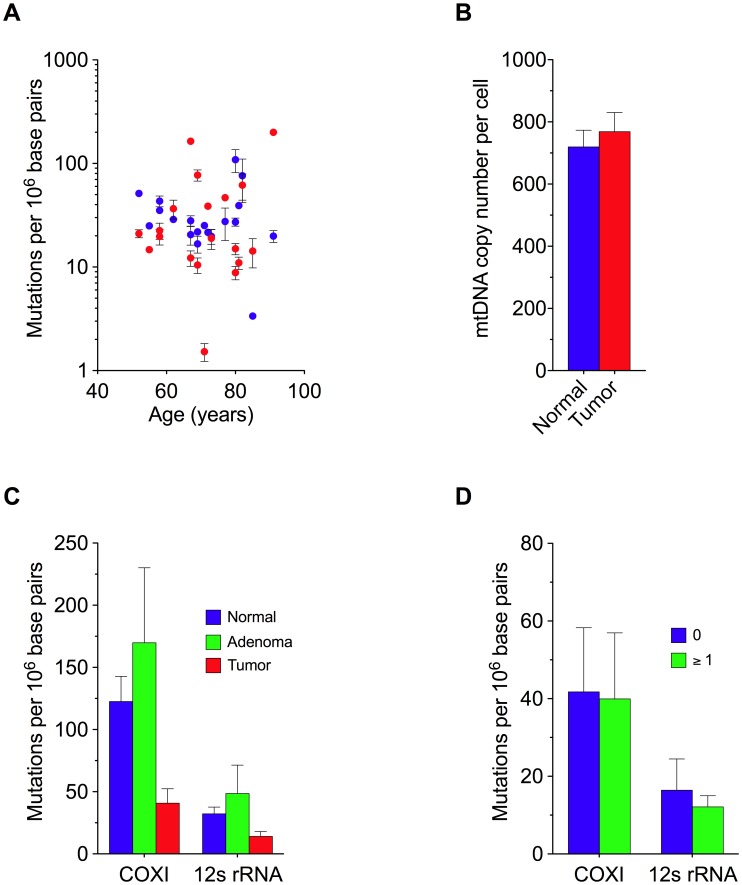
Random Mitochondrial DNA Mutations and Genome Copy Number in Colorectal Tissue. (A) Frequency of mitochondrial mutation as a function of age. Mutation frequency was determined at the 12S rRNA gene (blue) and COXI gene (red) in mtDNA isolated from normal human colorectal tissue. Each data point represents one patient. (B) Mitochondrial genome content (± s.e.m.) in normal and tumor tissue. (C) Average frequency of random mitochondrial mutations in colorectal tissue. Mean (± s.e.m.) mutation frequency within the 12S rRNA gene and COXI gene in mtDNA isolated from patient-matched normal (n = 20), adenoma (n = 8), and carcinoma (n = 20) colorectal tissues. (D) Patient-matched comparison of the mean (± s.e.m.) mtDNA random mutation frequency as stratified by carcinomas that harbored one or more clonal mutation.

Next, we quantified the frequency of random mutations in colorectal tissue using the RMC assay. We found that in normal colorectal tissue, mutations occurred at an average absolute frequency of 3.2±0.5×10^−5^ per base pair at a site in the 12S rRNA subunit (Figure 1A), and 1.2±0.2×10^−4^ at a second site in the COXI gene (Figure 1B). At both sites, mutation frequency was independent of patient age (Figure 2A). Unexpectedly, when we examined patient-matched colorectal carcinomas we found that, on average, tumor cells displayed an approximately three-fold lower random mutation frequency compared to normal colonic tissue (1.4±0.4×10^−5^, P = 0.004 for 12S rRNA, 4.1±1.2×10^−5^, P<0.001 for COXI, 2 tailed paired t-test) (Figure 1A–1B). No appreciable difference in mtDNA copy number was observed between the tissues (Figure 2B). These mtDNA findings are in stark contrast to previous observations in which we demonstrated a>100-fold increase in the frequency of random mutations in the nuclear DNA of human tumors [11].

The *frequency* of random mutations within a tissue is proportional to both the *rate* of *de novo* mutations per generation and the total *number* of cell divisions having occurred during development and aging. The observed decrease in mutation frequency within tumors could, thus, either be the result of a reduced mutation rate during tumor growth, or simply a consequence of the fact that the number of cell divisions having occurred since founding of the final clonal tumor outgrowth may be fewer than the number having occurred between development and sampling of normal adult colon tissue [12]. Genetic bottlenecking during the clonal formation of a carcinoma from a single founder cell effectively purges mutational diversity that has accumulated in normal colonic mucosa over a lifetime by repopulating it with closely related progeny [13].

To assess whether clonal expansion alone might account for the decreased mutation load associated with tumor mtDNA, we quantified the frequency of random mutations in adenoma tissues. Advanced adenomas are generally believed to be clonally derived [14], and the presence of clonal mtDNA mutations empirically demonstrates this to be true in at least 32% of our samples (Table 1). Given this, if clonal expansion alone were to underlie the decrease in random mutation burden, we would expect the frequency of random mutations in adenoma mtDNA (4.9±2.3×10^−5^ per base pair for 12S RNA, and 1.7±0.6×10^−4^ for the COXI gene) to be lower than that of patient-matched normal colon mtDNA (3.2±0.5×10^−5^ per base for 12S rRNA and 1.2±0.2×10^−4^ at the COXI gene), but this was not the case (Figure 2C) (P = 0.73 for 12S RNA and P = 0.15 for the COXI gene, 2-tailed paired t-test). Moreover, the decrease in the level of genetic heterogeneity in tumors was independent of whether or not the cancers bore a separate clonal mutation (Figure 2D). Thus, this leaves the possibility of a reduction in mtDNA mutation rate during late tumorigenesis.

**Figure 3:**
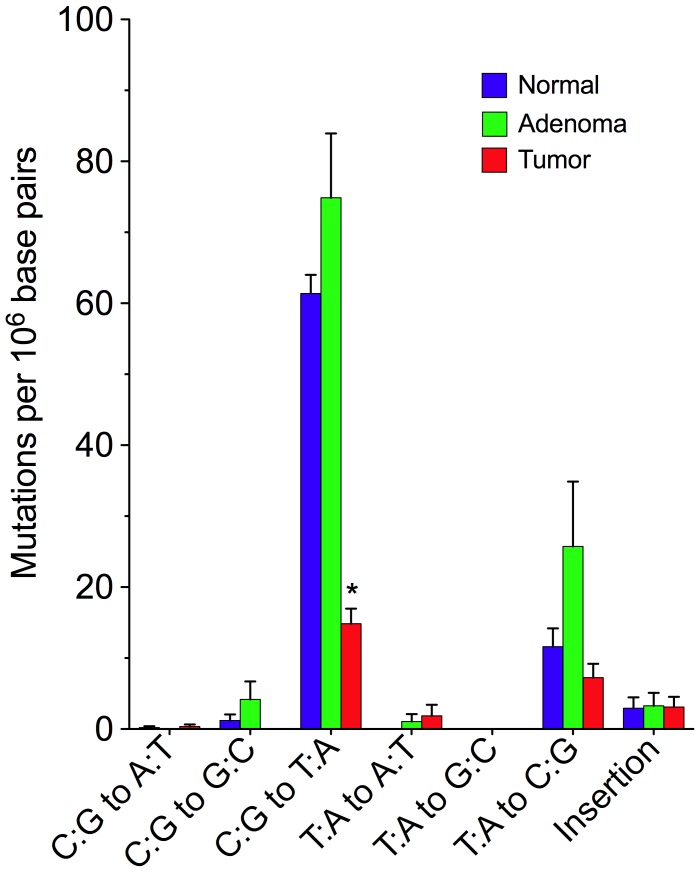
Decreased mtDNA Mutagenesis in Colorectal Carcinoma Is Attributable to a Reduction in C∶G to T∶A Transitions. Mitochondrial DNA mutation spectrum (± s.e.m.) per Mb in human colorectal tissue. Mutant mtDNA amplicons (n = 796) spanning two restriction sites (1215–1218 and 7335–7338) were recovered from the RMC assay and subjected to DNA sequencing to generate the mutational signature of mtDNA isolated from normal (n = 297), adenoma (n = 275), and carcinoma (n = 224) colorectal tissues. * *P*<0.0001; two-tailed paired t-test.

To gain further insight into possible mechanisms responsible for reducing mtDNA mutagenesis in tumor cells, we examined the mutation spectrum of 796 random mutation events (297 normal, 275 adenoma, 224 carcinoma). We found that in normal and adenoma tissues, C∶G to T∶A transitions predominated (Figure 3). Similar spectra have been previously reported in mice and are consistent with deamination of cytosine bases as the primary source of mutagenesis [10], a process that is driven by oxidative damage [15]. While all tissue types exhibited similar levels of T∶A to C∶G mutations, there was >3-fold decrease in C∶G to T∶A transitions among the tumor samples relative to normal colon, suggesting the source of these latter oxidatively-mediated mutations to be specifically reduced during carcinoma outgrowth. Interestingly, the majority of the clonally expanded mutations observed in carcinomas are C∶G to T∶A transitions (Table 1), which is similar to that previously reported in human colorectal tumors [16] and to the spectrum of random mutations in normal and adenoma tissue (Figure 3A), but is markedly different from the spectrum of mutations generated by polymerase γ on undamaged template [17], [18], [19]. This suggests that the biological change responsible for reducing the frequency of C∶G to T∶A mutations occurs after the initiation of neoplastic clonal expansion.

**Figure 4:**
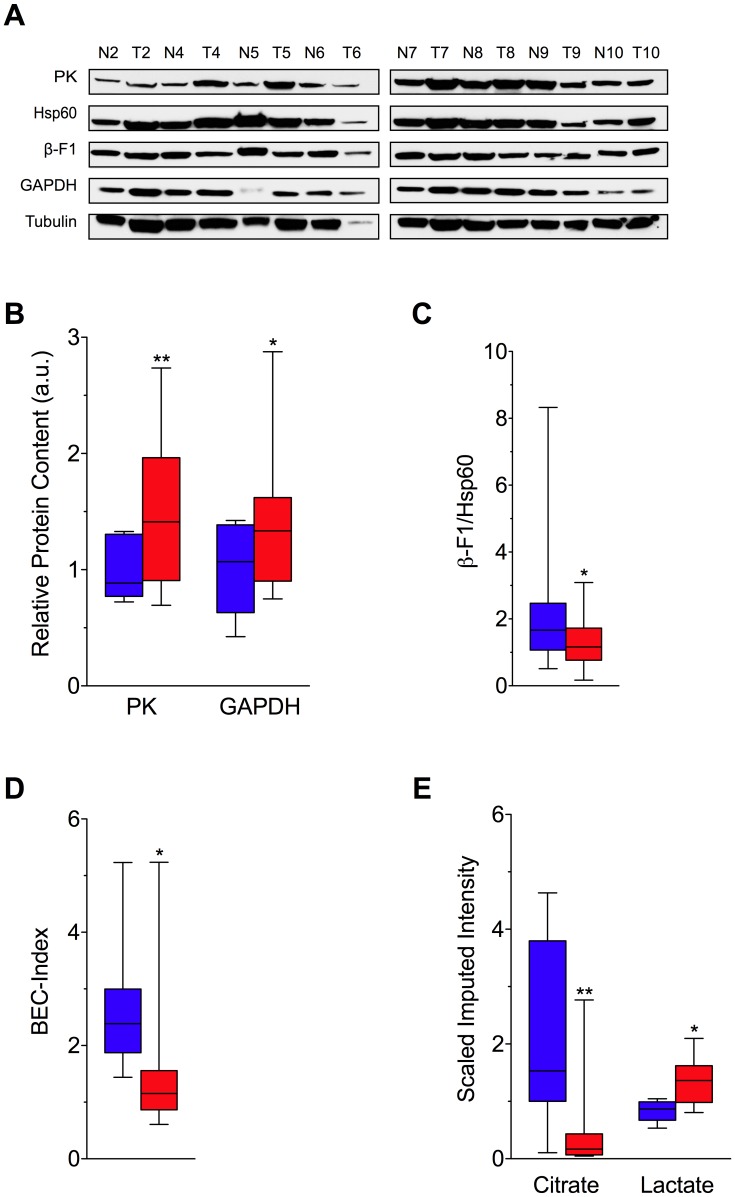
Metabolic Shift in Human Colorectal Cancer. (A) Western blot analysis of the expression levels of markers of oxidative phosphorylation (ß-F1-ATPase), structural function of the mitochondria (Hsp60), and the glycolytic pathway [GAPDH and pyruvate kinase (PK)], fractionated by SDS-PAGE and blotted with the corresponding antibodies from eight patient-matched normal (N) and tumor (T) human colorectal tissues. (B) The comparative cellular content of each glycolytic protein marker relative to the expression of tubulin in normal (blue) and tumor (red) tissue. (C) The bioenergetic competence of the mitochondria (ß-F1-ATPase/Hsp60 ratio) from tissues, and (D) overall mitochondrial potential of the cell, defined as the BioEnergetic Cellular Index (BEC index). BEC index is assessed by the ß-F1-ATPase/Hsp60/GAPDH ratio, providing a normalized proteomic evaluation of the metabolic shift in colorectal tumors. (E) Gas chromatography/mass spectrometry (GC/MS) metabolite analysis revealing significantly higher levels of lactate and lower levels of citrate in cancer tissues when compared to patient-matched normal colorectal tissue controls. Decreased citrate in tumors indicates reduced flux through the tricarboxylic acid cycle. Coupled with increased lactate levels, these metabolic alterations are consistent with a shift in glucose metabolism from oxidative phosphorylation (OXPHOS) to glycolysis among tumors. Box and whisker plots depict the median, distribution, and data range. The median is indicated by the black line, the box shows the interquartile range, and the ends of the whiskers the maxima and minima. * *P*<0.05; ** *P*<0.01.

One of the biochemical hallmarks of tumor cells involves the reprogramming of energy metabolism from primarily oxidative phosphorylation (OXPHOS) to anaerobic glycolysis [20], a phenomenon termed the Warburg effect [21]. This transition effectively decreases OXPHOS, and by extension, the production of reactive oxygen species (ROS) in the mitochondrial matrix, which have the potential to damage mtDNA [22]. To investigate whether the switch between oxidative phosphorylation and glycolysis is associated with a change in mtDNA mutation frequency, we analyzed the relative expression of protein markers for glycolysis and oxidative phosphorylation (Figure 4A). Consistent with an upregulation of glycolysis, we observed a significant increase in the glycolytic markers pyruvate kinase (PK) and glyceraldehyde 3-phosphate dehydrogenase (GAPDH) [23] in tumors relative to normal colon (Figure 4B). Moreover, as assessed by the abundance of the catalytic subunit OXPHOS marker ATP synthase (ß-F1-ATPase) [23], [24] normalized to the structural mitochondrial protein Hsp60, mitochondrial bioenergetic competence (i.e. OXPHOS) [25], [26], [27] is significantly decreased in colon carcinoma compared to normal controls (Figure 4C) (P<0.05, 2-tailed paired t-test). As such, the BioEnergetic Cellular (BEC) index, which gives the ratio of OXPHOS protein content to glycolytic protein content [26], [27], is reduced and consistent with a shift to Warburg metabolism in tumor tissue (Figure 4D) (P<0.05, 2-tailed paired t-test). In addition, to generate an instantaneous snapshot of the competing forms of glucose metabolism, we directly measured the relative amount of citrate and lactate in colorectal tissues by gas chromatography/mass spectrometry (GC/MS). We found that citrate, a tricarboxylic acid (TCA) cycle intermediate that correlates tightly with the level of mitochondrial respiration [28], [29], was significantly reduced in carcinomas relative to normal colon (Figure 4E) (P<0.01, 2-tailed paired t-test), whereas the level of lactate, the end product of anaerobic glycolysis [30], was increased (Figure 4E) (P<0.05, 2-tailed paired t-test).

**Figure 5:**
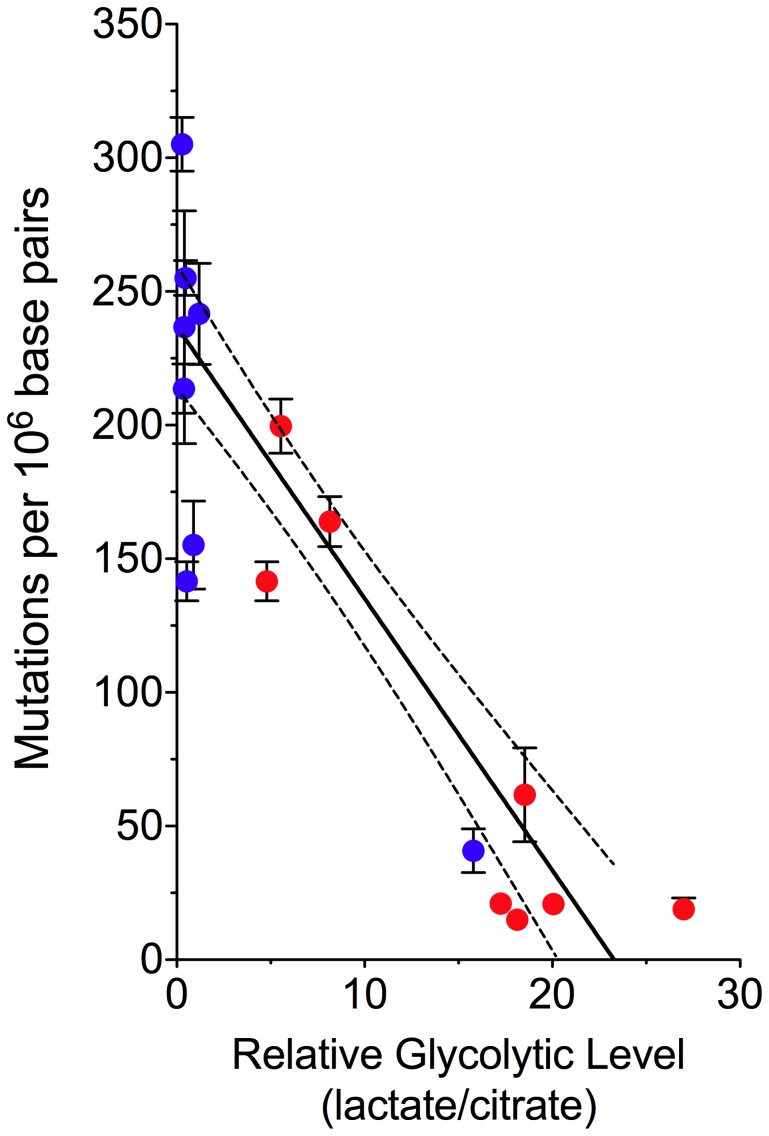
Decreased mtDNA Mutagenesis Is Coupled to a Shift in Glucose Metabolism. Normal (blue) and tumor (red) patient-matched colorectal tissue comparison of the mean mtDNA mutation burden (± s.e.m.) as a function of the tissue metabolic signature. This plot illustrates the inverse correlation between the level of mitochondrial respiration and mutagenesis (linear regression with 95% confidence intervals, slope −10.17±0.9894, significance of non-zero slope *P*<0.0001, R^2^ = 0.71).

Finally, to examine the relationship between energy metabolism and mtDNA mutation frequency, we plotted the random mutation frequency of all samples against the ratio of citrate to lactate (Figure 5). We found that in both normal and tumor tissue, mutation frequency decreases concomitantly with reduced mitochondrial respiration (linear regression: slope −10.17±0.9894, significance of non-zero slope P<0.0001, R^2^ = 0.71). This is consistent with the hypothesis that a large fraction of mtDNA mutagenesis is a consequence of oxidative damage generated as a byproduct during OXPHOS and provides a plausible rationale for the decreased frequency of random mutations in tumor cells–specifically, a decrease in C∶G to T∶A transitions (Figure 3), the most commonly observed mutations resulting from oxidative damage [15].

## Discussion

The experiments described here expose a surprising paradox in cancer biology. It has long been recognized that genomic instability is a hallmark of cancer cells. Whether instability is a driving force or an indirect consequence of cancer progression remains as contentious today as it was three decades ago [31], [32], [33]. The data presented above suggest that a mutator phenotype does not extend to the mitochondria and, in fact, mtDNA mutagenesis at the level of single base substitutions is suppressed within human colorectal tumors. While cells that acquire an increased rate of nuclear mutagenesis may be selected for to facilitate efficient generation of selectable genetic variants necessary for neoplastic evolution, no such pressures appear to exist for the mitochondrial genome.

Our data indicate that reduced mtDNA genetic diversity does not limit tumor progression, and that normal mtDNA mutation rates may actually serve as a barrier to cancer development. In support of this hypothesis is the large body of evidence espousing the mitochondrial theory of aging [34], [35], [36]. In this model, ROS production, mtDNA damage, mutation, and respiratory chain dysfunction are linked in a self-perpetuating cycle that generates a progressive decline of mitochondrial function, eventually impairing cell physiology and viability. In this context, a decrease in mtDNA mutagenesis secondary to a reduction in ROS might favor disease progression by breaking this cycle, and thereby contributing to the development of tumor cell immortality. By extension, it is conceivable that mitochondrial targeted cancer therapeutics focused on directly increasing mtDNA damage might efficiently suppress malignant growth. For example, reactivating tumor mitochondrial metabolism to increase mtDNA mutagenesis by pharmacologic measures, or developing mutagenic drugs with mtDNA trophism, might prove to be effective anticancer therapies.

## Materials and Methods

### Ethics statement

Ethical approval was granted by the St. Vincent's Hospital Ethics and Medical Research Committee to conduct all aspects of this study in accord with the principles expressed in the Declaration of Helsinki.

### Tissues and DNA isolation for mutational analysis

This study included 21 colorectal cancer patients (median age 72 years; range 52–85 years; 8 men, 13 women) treated surgically in the Centre for Colorectal Disease, St. Vincent's University Hospital, Dublin, Ireland. Tumor and matched normal mucosa were available for all cases. Tumors were staged using the WHO classification. There were 2 Stage I, 11 Stage II, 7 Stage III and 1 Stage IV cancers. 11/20 of these cases also had matched adenoma tissue. An additional 8 cases had adenoma and matched normal tissue (median age 69.5 years; range 59–83 years; 5 male, 3 female). Tissues collected were snap frozen and stored at −80 C.

Tissue samples (∼200 mg) were immersed in 5 mL homogenization medium (0.32 M sucrose, 1 mM EDTA, 10 mM Tris-HCl, pH 7.8) and disrupted with a glass Dounce-type homogenizer. The homogenate was transferred to a 15 mL tube and centrifuged at 1000 g for 10 min to isolate the nuclei and cell debris, followed by centrifugation of the supernatant at 13000 g for 20 min to pellet the mitochondria. The mitochondrial pellet was resuspended in 600 µL lysis buffer (10 mM Tris-HCl, pH 8.0, 150 mM NaCl, 20 mM EDTA, 1% SDS, and 0.2 mg/ml Proteinase K) and incubated at 55°C for 1 hr. MtDNA was isolated by phenol-chloroform extraction followed by isopropanol precipitation.

### mtDNA RMC assay

MtDNA was diluted and digested with 100 units *TaqI* restriction enzyme (New England Biolabs), 100 µg/mL BSA and a *TaqI*-specific digestion buffer (10 mM Tris-HCl, 10 mM MgCl2, 100 mM NaCl, pH 8.4) for 5 hrs, with 100 units *TaqI* added each hour. Mutation frequencies were determined for *TaqI-*digested mtDNA via real-time PCR in two separate reactions, one with a primer set flanking the *TaqI* restriction site to quantify the number of mutant molecules (i.e., those molecules resistant to digestion) and one with a set of primers in a region without a *TaqI* restriction site to quantify the total number of molecules. PCR was performed in triplicate in 25 µL reaction volumes, with 12.5 µL Brilliant SYBR Green QPCR Master Mix (Stratagene), 1 unit UDG (NEB), 1 µL of 25 µM forward and reverse primers, and diluted *TaqI-*digested mtDNA.

Mutant molecules were confirmed resistant to *TaqI* cleavage by post-PCR restriction digest and by sequencing. Amplicons were cloned into the pCR4-TOPO TA vector (Invitrogen) following the kit's instructions, and the resulting clones were transformed into DH5 alpha-T1R cells. The cells were plated on LB agar media supplemented with 50 µg/mL kanamycin and grown at 37°C overnight. Single colonies were picked into 1 mL LB media with 10% glycerol and incubated at 37°C for 16 hrs. These cultures were frozen and sent to the University of Washington High Throughput Genomics Center for rolling circle amplification and capillary sequencing.

### mtDNA copy number quantification

To obtain whole DNA (without mitochondrial enrichment), tissue samples (∼50 mg) were immersed in 5 mL homogenization medium (0.32 M sucrose, 1 mM EDTA, 10 mM Tris-HCl, pH 7.8) and disrupted with a glass Dounce-type homogenizer. The homogenate was transferred to a 15 mL tube and centrifuged at 13000 g for 20 minutes. The pellet was resuspended in 3 mL lysis buffer (10 mM Tris-HCl, pH 8.0, 150 mM NaCl, 20 mM EDTA, 1% SDS, and 0.2 mg/ml Proteinase K) and incubated at 55°C for 3 hr. DNA was isolated by phenol-chloroform extraction followed by isopropanol precipitation.

The mtDNA content per cell was determined via real-time PCR in two separate reactions, one with a primer set to mitochondrial DNA (ACCACAGTTTCATGCCCATCGT and TTTATGGGCTTTGGTGAGGGAGGT) to quantify mitochondrial genomes, and one with a primer set to the single copy nuclear ß-globin gene (GTGAAGGCTCATGGCAAGAAAG and TGTCACAGTGCAGCTCACTCAGT) to quantify nuclear genomes. Dilution curve controls demonstrated primer sets to be of equal amplification efficiency across a large range of template inputs. PCR was performed in duplicate in 25 µL reaction volumes, with 12.5 µL Brilliant SYBR Green QPCR Master Mix (Stratagene), 1 unit UDG (NEB), 1 µL of 25 µM forward and reverse primers, and diluted whole genomic DNA.

### Mitochondrial genome DNA sequencing

The entire mitochondrial genome was sequenced in the normal, adenoma, and carcinoma colorectal samples from each patient by first PCR amplifying the mtDNA with 28 pairs of primers, as previously described [37]. Clonally expanded mutations were scored only when the sequence of the adenoma or carcinoma mtDNA differed from that of the normal colorectal tissue. All regions with detected mutations were reamplified and sequenced to rule out the possibility of the mutations being produced by polymerase errors during the PCR or sequencing processes. In addition, to guard against the sample mix-up and contamination that has confounded many mtDNA mutation studies [38], we compared sequences of patient-matched tissues to the revised Cambridge Reference Sequence (rCRS) to confirm they shared common polymorphisms.

### SDS-PAGE and Western blot analysis

Total proteins were extracted from frozen tissue using an AllPrep DNA/RNA/Protein Kit (Qiagen) and dissolved in 8 M urea (Sigma). Protein preparations were diluted in Laemmli sample buffer (Bio-Rad; Hercules, CA) and subsequently incubated at 46°C for 10 min. Next, samples were loaded onto a 10% MiniProtean TGX precast gel (Bio-Rad) along with a protein marker (Bio-Rad). The gel was run with Tris-glycine-SDS buffer (Bio-Rad) for ∼38 min at 200 V, followed by blotting onto an Immun-Blot PVDF membrane (Bio-Rad) in Tris-glycine buffer (25 mM Tris, 192 mM glycine, 20% methanol, pH 8.3) for 60 min at 100 V at 4°C.

For Western blots, membranes were washed in TBST (TBS, 0.05% Tween 20) and incubated in blocking buffer (TBST containing 0.5% Tween 20 and 5% dried milk) for 45 min on a shaker. After blocking, the membranes were probed with primary antibodies at 4°C overnight. The membrane was washed and then incubated with secondary antibodies at room temperature for 1 h. Immunoreactive proteins were developed with the Super Signal West Pico enhanced chemiluminescence kit (Thermo Sci.) and exposed onto CL-XPosure Film (Thermo Sci.). Films were imaged with a CCD camera detection system and analyzed using Quantity One (Bio-Rad). Protein expression levels were defined as the sum of the intensities of the pixels inside the volume boundary×the area of a single pixel (in mm^2^), normalized with local background subtraction.

The following antibodies were used: goat polyclonal anti-Atp5B (C-20) (1∶ 1000, Santa Cruz Biotech; Santa Cruz, CA), mouse monoclonal anti-Hsp60 (H-1) (1∶1000, Santa Cruz Biotech), mouse monoclonal anti-GAPDH (6C5) (1∶3000, Abcam; Cambridge, MA), and goat polyclonal anti-pyruvate kinase (1∶1000, Abcam). Mouse horseradish peroxidase-conjugated anti-mouse and anti-goat were used as secondary antibodies (Vector Labs; Burlingame, CA). Tubulin was used as a loading control and was detected using a mouse monoclonal anti-ß-tubulin antibody (1∶1000, Invitrogen; Carlsbad, CA).

### Sample preparation for metabolic characterization

The sample preparation process was carried out using the automated MicroLab STAR system from Hamilton Company. Recovery standards were added prior to the first step in the extraction process for QC purposes. Sample preparation was conducted using a Metabolon proprietary series of organic and aqueous extractions to remove the protein fraction while allowing maximum recovery of small molecules. The resulting extract was divided into two fractions; one for analysis by LC and one for analysis by GC. Samples were placed briefly on a TurboVap (Zymark) to remove the organic solvent. Each sample was then frozen and dried under vacuum.

### Gas chromatography/mass spectrometry (GC/MS)

The samples destined for GC/MS analysis were re-dried under vacuum desiccation for a minimum of 24 hours prior to being derivatized under dried nitrogen using bistrimethyl-silyl-triflouroacetamide (BSTFA). The GC column was 5% phenyl and the temperature ramp was from 40° to 300°C in a 16 minute period. Samples were analyzed on a Thermo-Finnigan Trace DSQ fast-scanning single-quadrupole mass spectrometer using electron impact ionization. The instrument was tuned and calibrated for mass resolution and mass accuracy on a daily basis. The information output from the raw data files was automatically extracted as below.

### Bioinformatics

The informatics system consisted of four major components, the Laboratory Information Management System (LIMS), the data extraction and peak-identification software, data processing tools for QC and compound identification, and a collection of information interpretation and visualization tools for use by data analysts. The hardware and software foundations for these informatics components were the LAN backbone, and a database server running Oracle 10.2.0.1 Enterprise Edition.

### Metabolic compound identification

Compounds were identified by comparison to library entries of purified standards or recurrent unknown entities. Identification of known chemical entities was based on comparison to metabolomic library entries of purified standards. The combination of chromatographic properties and mass spectra gave an indication of a match to the specific compound or an isobaric entity.
